# Maximizing Degumming Efficiency for *Firmiana simplex* Bark Using Deep Eutectic Solvents

**DOI:** 10.3390/polym16152112

**Published:** 2024-07-24

**Authors:** Amjad Farooq, Muhammad Tauseef Khawar, Zongqian Wang, Mingwei Tian, Muhammad Mushtaq

**Affiliations:** 1 School of Textile and Garment, Qingdao University, Qingdao 266071, China; amjad@ahpu.edu.cn; 2 School of Textile and Garment, Anhui Polytechnic University, Wuhu 241000, China; 3 School of Engineering and Technology, National Textile University Faisalabad, Faisalabad 37610, Pakistan; tauseefkhawar@ntu.edu.pk; 4 School of Art and Design, National Textile University Faisalabad, Faisalabad 37610, Pakistan; mushtaqmalik@ntu.edu.pk

**Keywords:** deep eutectic solvents, degumming, choline chloride, urea, *firmiana simplex* bark

## Abstract

Degumming is a critical process in the purification of natural fibers, essential for enhancing their quality and usability across various applications. Traditional degumming methods employed for natural fibers encounter inherent limitations, encompassing prolonged procedures, excessive energy consumption, adverse environmental impact, and subpar efficiency. To address these challenges, a groundbreaking wave of degumming technique has emerged, transcending these constraints and heralding a new era of efficiency, sustainability, and eco-friendly techniques. This study represents the *Firmiana simplex* bark (FSB) fiber’s delignification by using deep eutectic solvents (DESs). The study explores the application of deep eutectic solvents, by synthesizing different types of DES using a hydrogen bond acceptor (HBA) and four representative hydrogen bond donors (HBDs) for FSB fiber degumming. This study investigates the morphologies, chemical compositions, crystallinities, and physical properties of *Firmiana simplex* bark fibers before and after the treatment. Furthermore, the effects and mechanisms of different DESs on dispersing FSB fibers were examined. The experimental results showed that choline chloride-urea (CU)-based DES initiates the degumming process by effectively disrupting the hydrogen bond interaction within FSB fibers, primarily by outcompeting chloride ions. Following this initial step, the DES acts by deprotonating phenolic hydroxyl groups and cleaving β-O-4 bonds present in diverse lignin units, thereby facilitating the efficient removal of lignin from the fibers. This innovative approach resulted in significantly higher degumming efficiency and ecofriendly as compared to traditional methods. Additionally, the results revealed that CU-based DES exhibits the utmost effectiveness in degumming FSB fibers. The optimal degumming conditions involve a precise processing temperature of 160 °C and a carefully controlled reaction time of 2 h yielding the most favorable outcomes. The present study presents a novel straightforward and environmentally friendly degumming method for *Firmiana simplex* bark, offering a substantial potential for enhancing the overall quality and usability of the resulting fibers. Our findings open new pathways for sustainable fiber-processing technologies.

## 1. Introduction

There are several excellent advantages associated with natural cellulosic fibers, which contribute to their widespread utilization. These fibers are derived from renewable sources, ensuring a sustainable and eco-friendly supply chain [1]. In response to consumer demand, researchers and industries are focusing on natural fibers such as kenaf, bast, jute, and a variety of agricultural waste materials [2,3,4]. Besides being widely known as the Chinese parasol tree or the Phoenix tree, also known as *Firmiana simplex*, it is one of the most prominent members of the Sterculiaceae family, and it is widely distributed in eastern Asia, from Okinawa to Honshu, as well as Taiwan, China, and several parts of Japan [5]. The leaves of *Firmiana simplex* are used to treat carbuncles, hemorrhoids, and sores with a lotion prepared from its roots [6]. Thus, *Firmiana simplex* leaves and roots have been the focus of many studies exploring its potential in traditional and modern medicines [7,8,9]. Nevertheless, the *Firmiana simplex* bark has never been explored for cellulose fiber extraction. This study addresses a critical gap in the research to investigate the potential of *Firmiana simplex* bark for cellulose fiber extraction. This novelty lies in evaluating a new source of cellulose fibers, which has been limitedly considered [5], thus opening new avenues for the utilization of this abundant natural resource. However, natural fibers require degumming before utilization in different industries, which is a process to remove the non-cellulosic content. Fiber degumming applications and the production of value-added products have been extensively investigated for natural fibers, including jute, ramie, kenaf, and hemp [10,11]. Plant fibers are degummed to eliminate their viscous or sticky materials from their surface, to enhance the efficiency of textile manufacturing industries.

The strong bonding between fibers caused by gummy constituents significantly hinders the extraction of fibers from natural sources. Several components can hinder fiber extraction, including hemicellulose, pectin, lignin, wax, and water-soluble substances. To ensure the utilization of cellulose fibers in diverse applications, a degumming process of *Firmiana simplex* bark fibers is necessary. However, there have been various degumming techniques before cellulose fiber applications [12,13] including acid, alkali, steam explosions, ultrasound, microwave, bacteria, fungi, and enzymes [14]. Despite this, these methods have significant drawbacks, including important environmental pollution caused by extensive usage of sodium hydroxide. Moreover, the traditional approaches suffer from limitations such as time-consuming procedures, expensive equipment, and high energy consumption, which hinder their further development [15]. Thus, exploring greener and more convenient alternatives for *Firmiana simplex* bark fiber’s degumming is imperative. Green solvents with low toxicity and high biocompatibility have emerged as prominent research areas in the field of pretreatment. Deep eutectic solvents have emerged as an outstanding category of green solvents, characterized by their composition comprising both hydrogen bond acceptors and hydrogen bond donors [16]. Various studies have been reported on using DESs to eliminate non-cellulosic impurities from biomasses. Choline chloride is widely used as HBA in the preparation of deep eutectic solvents for pretreatment [17]. Recently, other hydrogen bond acceptors are also being gradually explored by researchers [18]. Deep eutectic solvents have been confirmed to provide excellent features in lignocellulose pretreatment [19]. Some researchers have observed that deep eutectic solvents exhibit high solubility for lignin and hemicellulose during biomass fractionation processes [20]. However, the dissolution of cellulose in DES is seldom reported due to cellulose’s strong cohesive energy [21]. This selective removal capability for lignin and hemicellulose makes DESs promising for applications in natural-fiber degumming processes [22].

Song et al. [23] successfully utilized DESs for extracting fibers from Apocynum venetum as part of their degumming process. Similarly, Nie et al. [24] employed DESs in conjunction with steam explosion to separate kenaf fibers, while Yu et al. [25] applied DESs to pretreat raw ramie, subsequently obtaining cellulose nanofibrils. Despite these advancements, there remains a notable scarcity of the literature on DESs’ use in single-step separation of cellulose fibers from their sources. Moreover, the higher cost of DES reagents compared to traditional alkaline methods presents a significant barrier to widespread adoption. To overcome these challenges and enhance the sustainability of DES applications, it is crucial to further optimize extraction conditions. This approach aligns with efforts to promote a circular economy and sustainable development goals [26,27].

Thus, in this study, *Firmiana simplex* bark can be evaluated to prepare fibers by using deep eutectic solvents. By focusing on DESs, this study introduces an innovative and environmentally friendly method for degumming *Firmiana simplex* bark, highlighting the effectiveness of different types of DESs in removing non-cellulosic impurities and enhancing fiber quality. At present, the investigation of the degumming behavior of DES for natural fibers is very limited. However, according to limited previous studies on natural fiber degumming evaluation, results revealed that different types of DES have completely different effects on the removal of gummy components. Thus, degumming *of Firmiana simplex* bark was carried out and assessed to prepare fibers. Four different types of HBD-based DESs were synthesized while taking into consideration their nature as acids, amides, and polyalcohols. Thus, the novelty of this work is further emphasized by its potential to revolutionize the degumming process, making it more sustainable and efficient. This research not only provides a novel approach to fiber extraction from an underutilized natural resource but also contributes to the advancement of green chemistry by employing DESs, thereby aligning with global sustainability goals.

## 2. Materials and Methods

### 2.1. Materials

*Firmiana simplex* trees are widely available in China, the tree barks were provided by farmers from Jianhe City in Guizhou province, without any treatment. Sodium hydroxide (NaOH, 95%), choline chloride (CC, ≥98%), oxalic acid (OA, ≥98%), lactic acid (LA, ≥99.5%), urea (U, ≥99.5%), ethylene glycol (EG, ≥99.5%), acetone (≥96.5%), and sulfuric acid (H_2_SO_4_, ≥98.5%) were provided by Sinopharm Group Co., Ltd., Shanghai, China.

### 2.2. Methods

#### 2.2.1. Preparation of Deep Eutectic Solvents

Four hydrogen bond donors were selected to synthesize different types of DESs to evaluate the influence of HBDs on the degumming of *Firmiana simplex* bark. Deep eutectic solvents containing choline chloride–urea (CU), choline chloride–ethylene glycol (CE), choline chloride–lactic acid (CLA), and choline chloride–oxalic acid (CO) were prepared according to a standard method [28] by mixing at different molar ratios (1:2, 1:2, 1:2, and 1:1, respectively) at 80 °C to prepare acidic and basic DES solutions as mentioned in a previous study [29]. The mixtures were stirred until transparent solutions were obtained.

#### 2.2.2. *Firmiana Simplex* Bark Fiber Preparation

Based on several previous studies, we used standard methods to extract long fibers from *Firmiana simplex* bark [30,31]. The tree barks were cut into 7–10 cm lengths and rinsed with clean water to remove all contamination as shown in Figure S1. Finally, the prepared tree barks were soaked in freshwater (at 25 °C) for approximately 10 days to activate microbial degradation [32,33,34], to break the bonds between *Firmiana simplex* bark fibers.

#### 2.2.3. DES Treatment with *Firmiana Simplex* Bark Fibers

To analyze the effectiveness of various types of deep eutectic solvents, the *Firmiana simplex* bark fibers (1 g) were treated with DES (20 g) at specific reaction conditions including temperature at 140 °C for 2 h. The FSB fibers were treated in three different conditions: (1) the samples were taken and directly treated with DES, (2) FSB fibers were manually peeled from the bark and treated with DES, (3) collected FSB fibers after microbial degradation were heated with DES to analyze the degumming efficiency. At the end of pretreatment, the pretreated fibers were washed, and DES elimination was performed by using vacuum filtration. The conductivity of the wash water was measured, and if it was ≤20 μS/cm, the DES residue on the fiber sample was deemed to be effectively removed [35], and drying of the final product was performed. Finally, after evaluating the fiber yield and degumming efficiency of four different deep eutectic solvents, one particular DES was selected for further optimization. 

### 2.3. Characterization

The chemical composition of the FSB fibers was estimated for cellulose, hemicellulose, and lignin content by using the standard gravimetric method [36] as used in our previous study for a chemical compositional analysis of *Ficus natalensis* bark [37], and the Conrad technique was used to evaluate wax content in the FSB fibers. Additionally, ASTM D 2654-67 [38] and ASTME1755-01 [39] standard methods were used to estimate moisture and ash content, respectively [37,40,41]. The surface morphologies of raw material and the obtained fibers were analyzed by using a scanning electron microscope (SEM) (S-4800, Hitachi, Tokyo, Japan) with different magnification levels at a specific accelerating voltage (5.0 kV). Before SEM testing, the surface of the samples was coated with gold deposit in a vacuum for 1 min. The yield of degummed fiber was evaluated according to Equation (1) as mentioned in a recent study [42,43]:

(1) $$Yield(\%)=\frac{G0}{G}\times 100$$

where G0 shows the dry weight of fibers after DES pretreatment and G shows the weight of dry fibers before DES pretreatment.

The residual gum content of the FSB fibers was evaluated through China National Standard GB 5889-1986 [44] by using Equation (2) as mentioned in [45]. Briefly, fiber samples (5 g) were oven-dried to constant weight; later on, samples were treated with 20 g/L NaOH solution (100 mL) for 3 h. After the reaction, the fiber samples were finally dried again to a constant weight.

(2) $$Residual\;gum\;content\;(\%)=\frac{G0-G}{G}\times 100$$

where G0 is the dry weight of the FSB fibers and G is the dry weight of the fibers after being cooked in NaOH (20 g/L) solution.

The Chinese standard (GB/T 18147.5-2015 [46]) was used to evaluate the physical and mechanical properties of the fibers. Before testing, samples were carefully placed under standard conditions by maintaining the temperature at 20 ± 2 °C and relative humidity at 65 ± 2% for 24 h. The linear density (dtex) and tenacity (cN/d) were calculated by Equations (3) and (4), respectively.

N = 10 G/nL(3)

where G shows the weight (g) of fiber, n is the number of fibers, and L is the cut length of fiber samples (20 mm).

T = F/Ndtex(4)

where F is the breaking strength (cN), and Ndtex is the linear density.

Fourier-transform infrared spectrometer (FTIR) was used (Prestige-21, Shimadzu, Kyoto, Japan) for possible changes in the structure of pretreated fibers. For each sample, the transmittance of the infrared was recorded from 600 to 4000 cm^−1^ at a specific resolution (2.0 cm^−1^) for infrared spectra. X-ray diffraction (XRD) was used to measure the crystallinity index (CrI) of the fiber samples by using an XRD machine (D8, Bruker, Karlsruhe, Germany). Using Segal’s method, the CrI was calculated by subtracting the minimum intensity at 18.6° from the maximum peak intensity at 22.6° and dividing the result by the maximum peak intensity [1].

## 3. Results and Discussion

Following pretreatment with various DESs, an evaluation was conducted to identify the most suitable solvent for the degumming of FSB fibers. Among the tested solvents, the best DES solvent with notable characteristics in terms of degumming efficiency, residual gum content, preservation of fiber structure, and mechanical properties was selected. Consequently, based on these comprehensive parameters, the selected DES was chosen for further detailed analysis, while other DES pretreatments were not selected for subsequent investigation.

### 3.1. Chemical Composition Analysis

The chemical composition of *Firmiana simplex* bark was investigated by using standard methods and the applied procedure as detailed in Supplementary Materials. The FSB contains a higher amount of cellulose at 71.12 ± 1.5%, hemicellulose at 13.11 ± 0.9%, and non-cellulosic compounds at 16.64 ± 1.03% (lignin) as recently mentioned [5]. The higher fiber content in FSB shows the feasibility of extraction of cellulose and nanocellulose. The hydrophilic characteristics of FSB fibers are significantly influenced by the presence of lignin, which accounts for approximately 16.34 ± 1.03% of the composition. Additionally, a specific wax percentage (0.91 ± 0.03%) enhances the fiber’s surface roughness properties by promoting fiber adhesion. Several factors, including plantation conditions, plant age, and soil conditions influence the moisture content of FSB fibers. Consequently, the fibers’ moisture content was measured as 11.5 ± 0.09%. A comparative chemical composition analysis has been shown in Table S1, indicating a higher amount of cellulose content than many other sources. Table S1 shows that cellulose is the main constituent in most biomasses, usually outweighing the total amount of hemicellulose and lignin by more than two times. Variability in extractives, moisture, wax, and pectin content across different species can cause total values of cellulose + hemicellulose + lignin to vary at around 100%. The results further show that the fiber sources mentioned in Table S1 can be categorized into two main groups: the majority, characterized by high cellulose content but low hemicellulose and lignin content similar to typical non-bark textile fibers like hemp fibers [47], and a second group including fibers such as Napier grass and corn hub, where cellulose comprises less than 50% of the total fiber content. The cellulose content of FSB fibers is similar to that of fibers derived from various other bark sources, such as Pithecellobium dulce and Ficus racemose, exhibiting cellulose contents of 75.15% and 72.36%, respectively.

### 3.2. Morphological Analysis

The morphological analysis of raw FSB fibers and degummed fibers treated with DESs was systematically investigated. First, the as-received sun-dried tree barks were directly treated with various solutions of DESs at the precise reaction conditions using an oil bath system. Photographs of unpeeled fibers from raw bark and degummed fibers isolated by various types of DESs are shown in Figure S2. The dispersion of unpeeled fibers treated with every type of DES is not obvious because of robust fiber networks as shown in Figures S2 and S3. Next, after chopping and manual peeling the bark tree fibers, the impact of different DESs was also not clear because of the intensity of the fibers connected strongly with one another (Figure S3).

**Figure 1:**
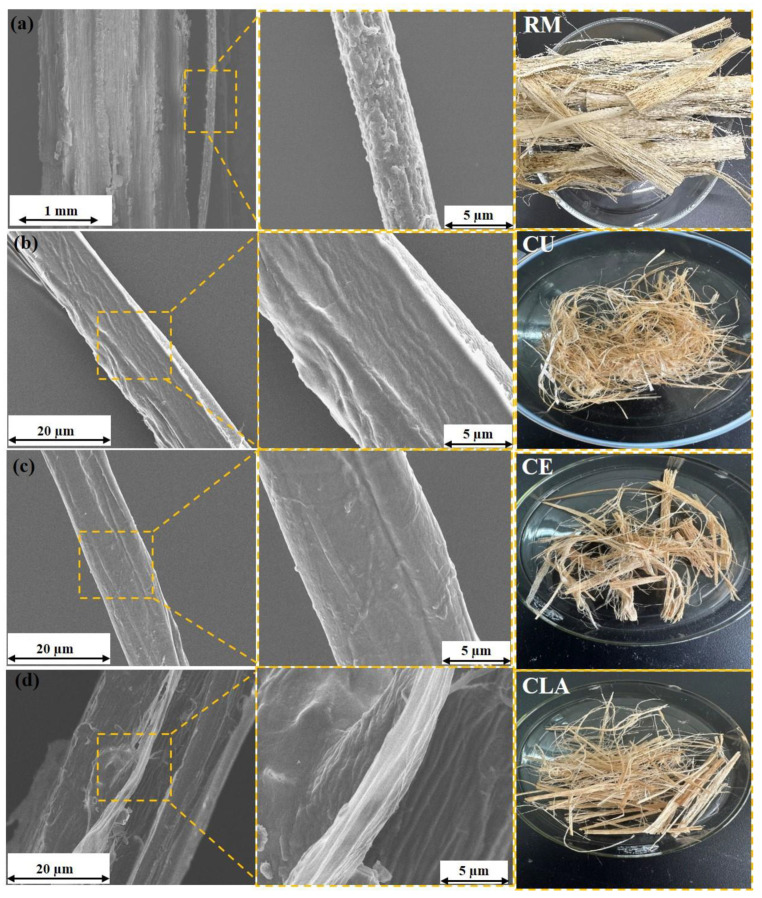
Manually peeled raw material (RM) water-retted fibers (**a**), fiber samples pretreated with CU (**b**), CE (**c**), and CLA (**d**).

**Figure 2:**
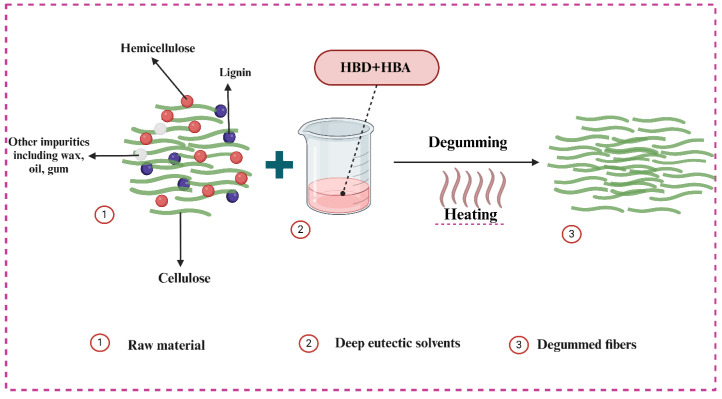
Possible degumming mechanism of FSB fibers.

Finally, the water-retted bark fibers obtained after manual peeling were treated and analyzed. Distinct dispersion and degumming effects were observed with different DES types (CE, CLA, CO, and CU). The effects of DES based on CU, CLA, and CE on FSB bark fibers were further investigated by using SEM morphology. Initially, as shown in Figure 1a, the raw bark fibers do not appear as single fibers because of the presence of gummy material among the internal and external structures of the fibers. During the degumming process, the gummy material coated on the surface of the bark dissolves easily in the deep eutectic solvent and is eliminated during subsequent washing. The physical appearance of raw water-retted bark and fibers prepared after treatment with various DESs is shown in Figure S4. The raw material, covered with a thick layer of gum, appears darker in color and has a relatively hard texture (Figure 1a and Figure S4a). Chemical treatments successfully remove surface contaminants and non-cellulosic components, resulting in bark fiber defibrillation (Figure 2) [48]. Additionally, it improves the clarity of cellulose fibers by successfully eliminating non-cellulosic impurities, giving them a distinctive, refined appearance. However, fibers treated with various DESs exhibited a softer texture and lighter color, as depicted in Figure 1b–d. These changes in fiber structure suggest successful dissolution and removal of gummy material. SEM images illustrate that fibers treated with CU appeared smooth and soft, devoid of any residual degumming material (Figure 1b) as also shown in the physical appearance of the samples in Figure S4e. The dispersion of CE is not very obvious at a temperature of 140 °C for 2 h as shown in Figure 1c; similar findings have also been observed where polyol-based deep eutectic solvents show a limited effect on the delignification of biomass materials (Figure S4c) [49,50]. Because of the formation of strong hydrogen bonds between polyol-based deep eutectic solvent molecules, generally, they need more severe reaction conditions including a high reaction temperature and long reaction time [51,52]. In contrast, treatment with CLA resulted in surface disruptions (Figure 1d), indicating partial fiber degradation likely due to the acidic nature of the solvent [53].

The influence of lactic acid- and choline chloride-based DES on fiber properties was obvious; a slight destruction in the fiber’s structure was observed which was also evidenced in the lower mechanical properties of these samples. It was the acidic impact of the solvent that made them weak at some points. However, FSB fibers pretreated with CO were completely dissolved into the solution (Figure S4d). The observed increase in fiber dissolution in DES solution indicated the degradation of cellulose, which was more pronounced with CO, suggesting the breaking of molecular chains of cellulose in amorphous and crystalline regions [54]. CO-based DES was reported to degrade some oligosaccharides and a portion of amorphous cellulose, thereby loosening the fiber structure as found in a previous study [55]. Finally, from these results, eutectic solvents based on oxalic acids were considered not suitable for FSB fiber degumming at specific reaction conditions. Figure 2 illustrates that the fiber structure undergoes significant dispersion upon treatment with CU, CLA, and CE, indicating the removal of a majority of the non-cellulosic constituents during this process.

### 3.3. Residual Gum Content

**Figure 3:**
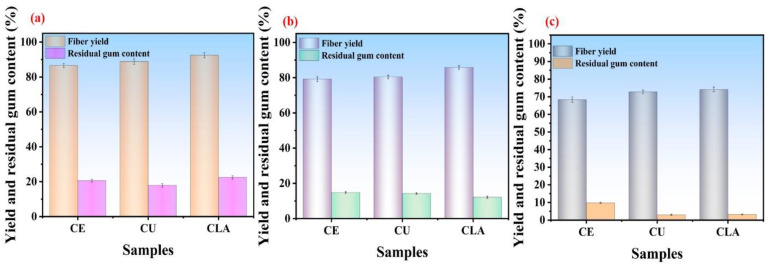
Fiber yield and residual gum content values obtained after the degumming process and heated by various DESs at different reaction conditions: DES degumming of raw bark without chopping of bark fibers (**a**), fibers when manually chopped and peeled and degummed with different DESs (**b**), and degumming after water-retting process (**c**).

The sun-dried FSB fibers were initially cut into small pieces and directly treated with various types of DES. The solid-to-liquid ratio was taken as 1:20, and the temperature was set at 140 °C with a fixed reaction time of 2 h. The degumming process of FSB fibers (directly pretreated without any fiber treatment) resulted in fiber yields of 92.48 ± 1.5%, 86.61 ± 1.2%, and 88.81 ± 1.6% for CU, CE, and CLA, respectively. Higher residual gum contents were observed at 20.6 ± 0.8%, 22.52 ± 0.9%, and 17.9 ± 1.03% for CE, CLA, and CU, respectively, as depicted in Figure 3a. The results revealed that residual gum content is high for every sample which means that the removal of non-cellulosic impurities was not effectively performed; thus, changes in the FSB fiber raw materials were made. For example, due to the initial hardness of the bark fibers, subsequent experiments involved manually peeling and separating the bark fiber layers by hand, while keeping time, temperature, and the solid-to-liquid molar ratio constant. The fibers were then treated again under identical conditions. This modification slightly reduced the degumming efficiency compared to the treatment of un-chopped bark fibers, resulting in fiber yield efficiencies of 85.8 ± 1.02%, 80.4 ± 1.04%, and 79.2 ± 1.3% for CLA, CU, and CE, respectively (Figure 3b). The residual gum content was similarly observed to be 14.9 ± 0.456%, 14.3 ± 0.4%, and 12.2 ± 0.56% for CE, CU, and CLA, respectively, across the various DES treatments. However, the higher values of residual gum content still show that this pretreatment technique did not eliminate non-cellulosic impurities.

Then, finally, the FSB fibers were water-retted, and chemical heating of the fibers was performed under the same reaction conditions as shown in Figure S1. The degumming efficiency and residual gum content of the water-retted FSB fibers were carefully evaluated. The results indicated that the fibers treated with deep eutectic solvents achieved excellent degumming, with significantly reduced residual gum content. The appearance of the degummed fibers closely resembled that of cellulose fibers, having a cellulose content comparable to the original FSB bark (71.12 ± 1.5%, Table S1). The water-retted treatment of fibers was deemed highly effective, demonstrating the suitability of various types of DES. Specifically, fiber yields were measured at 68.4 ± 1.5%, 74.2 ± 1.3%, and 72.8 ± 1% for CE, CLA, and CU, respectively. Residual gum content was notably low, with values of 9.83 ± 0.36%, 3.03 ± 0.3%, and 3.24 ± 0.2% for CE, CLA, and CU, respectively, as shown in Figure 3c.

CLA exhibited exceptional fiber degumming efficiency and minimal residual gum content. However, the SEM analysis revealed structural disruptions and surface damage to the fibers (Figure 1d), findings corroborated by mechanical characterization. In contrast, fibers treated with choline chloride and urea showed slightly lower degumming efficiency and a comparable residual gum content compared to those treated with choline chloride and lactic acid-based DES. Nevertheless, CU-based DES samples by SEM imaging (Figure 1b) demonstrated a smooth fiber structure and excellent surface characteristics. Additionally, these fibers exhibited superior mechanical properties, highlighting the effective degumming efficiency achieved by choline chloride and urea. Based on these findings, choline chloride and urea were identified as optimal deep eutectic solvents for FSB fiber degumming. The reaction parameters were optimized to a solid-to-liquid molar ratio of 1:40, temperature of 140 °C, and duration of 2 h.

### 3.4. Chemical Structural Changes and Mechanical Properties Analysis

**Figure 4:**
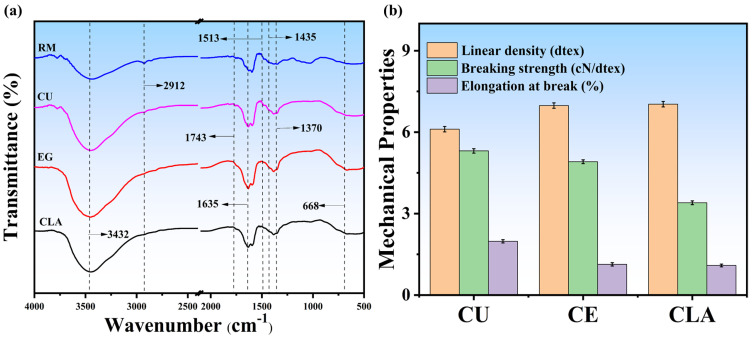
FTIR spectra (**a**) and mechanical properties (**b**) of raw material (Rm) and different fiber samples pretreated with DESs.

The chemical structural changes in the fibers were further verified by FTIR spectra. FTIR spectra of FSB fiber raw material have also been studied in our recent study [5]. The characteristic peaks of cellulose were found in all samples, including stretching vibrations of –OH at 3432 cm^−1^, stretching vibrations of C–H at 2912 cm^−1^, and bending vibrations of C–OH at 668 cm^−1^ [13,56] as shown in Figure 4a. This indicates that the structure of cellulose did not change after degumming. Additionally, a decrease in peak intensity at 2912 cm^−1^ occurred when deep eutectic solvents were applied to the raw fibers, probably caused by the dissolution of hydroxyl groups in lignin and partial cellulose decomposition during the degumming process. Similarly, the increased intensity of the peaks at 1370 cm^−1^ shows the presence of cellulose and the removal of non-cellulosic impurities [57]. According to Meng et al. [58], the band at 1743 cm^−1^ is caused by C–OH bending and C–O stretching from hemicellulose. However, the peak intensity was strongly reduced or disappeared (samples treated with DES); this indicates the capability of DESs to remove hemicellulose from raw fibers. According to Klall et al. [59], lignin functions as an aromatic skeleton (1513 cm^−1^), benzene stretching ring (1635 cm^−1^), and O–CH_3_ of the benzene ring (1435 cm^−1^) (benzene stretching ring); however, the reduction in the intensities of characteristic lignin peaks of the treated samples indicates that the DES is highly effective in removing the lignin from the fiber samples. Additionally, deep eutectic solvent-treated fibers were further analyzed by evaluating mechanical properties such as linear density, breaking strength, and elongation at the break of all the samples as detailed in Table S2. Fibers treated with choline chloride- and urea-based deep eutectic solvents displayed excellent breaking tenacity and linear density values as compared to the other samples exhibiting 5.31 ± 0.08 cN/dtex and 6.11 ± 0.1 dtex, respectively. Additionally, for the CU fiber samples, the elongation at break was noted as 1.98 ± 0.05%. The mechanical properties of the CE-treated samples revealed comparatively lower values of breaking tenacity and linear density exhibiting 4.91 ± 0.07 cN/dtex and 6.98 ± 0.1 dtex, respectively, and elongation at break was noted as 1.13 ± 0.05% (Figure 4b). As a result of severe fiber damage during DES pretreatment as revealed by SEM morphology, the samples treated with CLA had lower mechanical properties (breaking strength 3.40 ± 0.07 cN/dtex, linear density 7.03 ± 0.1 dtex, and elongation at break 1.09 ± 0.05 %) than the other samples. As a result of CU treatment, the fibers’ tenacity (Grade 2 ≥ 4.00 cN/dtex) and linear density (Grade 3 ≤ 8.33 dtex) meet China’s national standards for textiles (GB/T 20793-2015) [60].

Choline chloride- and urea-based deep eutectic solvents have proven to be superior for treating FSB fibers. This superiority is evidenced by their excellent mechanical properties, including high breaking tenacity (5.31 ± 0.08 cN/dtex), optimal linear density (6.11 ± 0.1 dtex), and favorable elongation at break (1.98 ± 0.05%). Furthermore, the SEM morphology analysis revealed that CU-treated fibers exhibited smooth, soft surfaces devoid of residual degumming material, indicating minimal fiber damage. In contrast, the fibers treated with other DESs, such as CLA, showed significant degradation and inferior mechanical properties. Based on these findings, choline chloride and urea were identified as optimal deep eutectic solvents for FSB fiber degumming. Therefore, CU-DES stands out as the most effective solvent for further investigations into fiber treatment applications.

Thus, CU-DES was further investigated by pretreating the FSB fibers at different times and temperatures to optimize the degumming process.

### 3.5. Optimization Analysis of CU-Based DES Treatment

#### 3.5.1. Influence of CU-Based DES Treatment on Fiber Yield and Residual Gum Content

**Figure 5:**
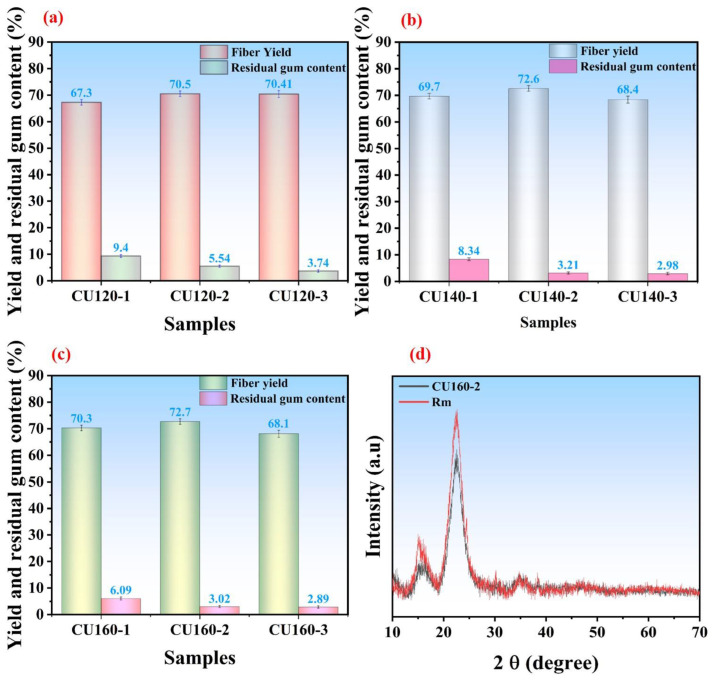
Fiber yield and residual gum content of CU-based DES pretreated fibers at 120 °C (**a**), 140 °C (**b**), and 160 °C (**c**) at different time cycles (1, 2, and 3 h). XRD spectra of raw fiber materials (RM) and CU pretreated fiber sample (**d**).

To investigate further and optimize the reaction conditions, the FSB fibers were further treated with CU-based DES at different times and temperatures. The degumming of bark tree fibers was conducted in a single-step process without combining additional treatments. Various durations (1, 2, and 3 h) and temperature ranges (120, 140, and 160 °C) were tested to optimize reaction conditions using deep eutectic solvents, specifically choline chloride and urea. The nomenclature of the samples reflects these conditions (e.g., CU120-1 for 120 °C and 1 h, CU140-2 for 140 °C and 2 h, and so on). The results indicated that as the temperature increased, the yield of the degummed fibers initially rose but then decreased after prolonged exposure at higher temperatures. For instance, yields were recorded as 70.41 ± 1.3%, 68.4 ± 1.3%, and 68.1 ± 1.3% for 3 h at 120 °C, 140 °C, and 160 °C, respectively (Figure 5a). Detailed results are provided in Table S3.

Similarly, residual gum content decreased with higher temperatures, showing values of 3.74 ± 0.45%, 2.98 ± 0.45%, and 2.89 ± 0.45% at 120 °C, 140 °C, and 160 °C, respectively (Figure 5a). Choline chloride and urea-treated fibers exhibited a maximum yield of 72.7 ± 1.04% at 160 °C for 2 h, with residual gum content at 3.02 ± 0.4% (Figure 5c). At 140 °C for 2 h, the degumming efficiency was 72.6 ± 1.04% with a residual gum content of 3.21 ± 0.4%. However, for 1 h at 120 °C, 140 °C, and 160 °C, degumming efficiencies were 67.3 ± 1.02%, 69.7 ± 1.02%, and 70.4 ± 1.3%, respectively (Figure 5b). Notably, the 70.3 ± 1.02% efficiency at 160 °C for 1 h was comparable to the values at 120 °C for 2 h (70.5 ± 1.04%) and 3 h (70.4 ± 1.3%) but with a higher residual gum content (6.09 ± 0.56%), indicating incomplete removal of non-cellulosic materials due to the shorter reaction times.

Based on these experiments, the optimal conditions for degumming FSB fibers were determined as 160 °C for 2 h, balancing high degumming efficiency and minimal residual gum content. The crystallinity (CrI) of the fiber samples was measured before (raw material) and after treatments (CU160-2) were measured which shows the physicochemical characteristics of fiber materials. The two selected fibers reveal the typical cellulose structural pattern exhibiting diffraction peaks approximately at 15° and 22.6°, which shows a correspondence of 1–10 and 200 lattice planes, respectively [61,62], as depicted in Figure 5d. Similar findings have also been observed in our recent research work [5]. The crystallinity index was obtained from a height of (200) peaks and a minimum intensity between (200) and (110) peaks. The crystallinity index of water-retted raw material was noted approximately as 64.9%; however, it increases to 70.12% for the sample CU160-2, which shows that choline chloride- and urea-based DESs were able to eliminate a large proportion of hemicellulose and lignin. However, the distinct peaks for cellulose show that cellulose was not removed during CU pretreatment.

#### 3.5.2. Morphological Analysis and Degumming Mechanism of DES

**Figure 6:**
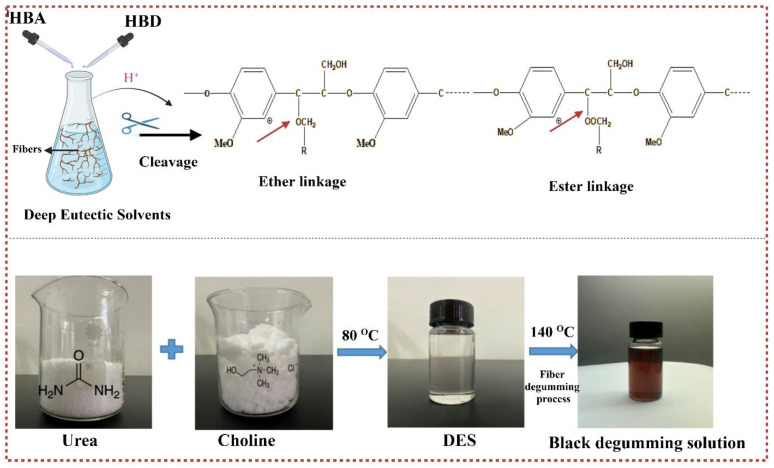
Degumming of DES initiated by the cleavage of ether and ester bonds within FSB fibers enabled by DES.

According to fiber degumming efficiency and residual gum content values of choline chloride and urea, the four best samples were selected, and morphological analyses were performed. The results revealed that the influence of reaction conditions including time and temperature impacted the structural appearance and the yield of the obtained fibers. The selected SEM images revealed that the non-cellulosic impurities were removed effectively using CU-based deep eutectic solvents as the surface of all the samples is smooth and soft. The results indicate that CU-based DES treatments can remove most of the gummy material within the raw material (Figure S5a). However, sample CU120-2 exhibited groves and hardness on the surface of the fibers as compared to the other samples, possibly because of its higher gum content (Figure S5b). The fibers treated at CU160-2 as shown in Figure S5c show that the gummy material has been removed as the fiber surface is uniform and soft as compared to samples CU120-1 and CU160-3 (Figure S5b,d). However, at higher temperatures and long reaction times, the obtained fiber morphology was not effective because of the destruction of the fiber’s surface, for example, sample CU160-3 as shown in Figure S5d. Hemicellulose and lignin are strongly linked within the natural fiber by covalent bonds known as α-benzyl ether linkage and physical intermixing. Additionally, they are tightly interconnected with cellulose through very strong hydrogen bonds, forming a rigid structure of lignin, hemicellulose, and cellulose [12,63,64,65]. The significant efficient process involved in deep eutectic solvent fractionation is the different dissolution abilities of three significant constituents: cellulose, hemicellulose, and lignin [66]. However, deep eutectic solvent treatment disrupts the hydrogen-bonding interaction in FSB fibers. It occurs due to chloride ions (present in DES) competing with the hydroxyl groups in carbohydrates and lignin for hydrogen bond formation, dismantling the linkage between lignin–carbohydrate complexes [67]. The dissolution of lignin is facilitated by DES breaking the ester and ether bonds of lignin [23] as shown in Figure 6. Additionally, the proton (H^+^) separated from the hydrogen bond donor acts as a catalyst, enabling the cleavage of ether and ester bonds in lignin and hemicellulose. Subsequently, it breaks hemicellulose and lignin, quickly enhancing their solubility in deep eutectic solvents [68] (Figure 6). Initially, the degumming solution exhibited a colorless and transparent appearance. However, it underwent a notable transformation turning into a black color. This color change is attributed to the dissolution of pectin, lignin, hemicellulose, and other low-molecular-weight polymers. Figure 6 visually represents this transformation. The use of a urea-based deep eutectic solvent (CU-DES) in this study can be correlated to the specific chemical composition of the fibers, characterized by high cellulose content and low lignin content (Table S1). This composition necessitates milder treatment conditions for effective lignin removal without compromising the structural integrity of the fibers, as compared to traditional pretreatment techniques [26]. Urea, as a component of the DES, plays a critical role in the dissolution process by disrupting the hydrogen-bonding network between cellulose, hemicellulose, and lignin. The mild yet effective action of CU-DES ensures that the non-cellulosic impurities are removed efficiently while preserving the cellulose structure. This selective dissolution capability makes CU-DES a suitable and efficient solvent for the degumming and purification of natural fibers, as demonstrated by the smooth and soft surfaces of the treated fibers in our SEM analysis. Hence, the suitability of CU-DES for this research is underpinned by its ability to provide the necessary mild conditions for lignin removal and fiber purification, aligning well with the chemical nature of the raw materials used.

#### 3.5.3. Chemical Composition after CU-Based DES Pretreatment

**Figure 7:**
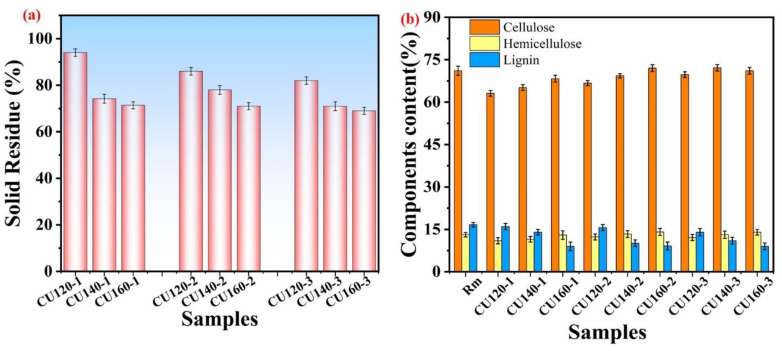
Solid residue content (**a**) and residual components obtained after CU-based pretreatment (**b**).

The chemical composition, removal of non-cellulosic impurities, and solid residues before and after treatment with choline chloride- and urea-based deep eutectic solvents were analyzed for FSB fibers. Following DES pretreatment, a decrease in solid residues was observed with increasing reaction temperature, indicating enhanced degumming efficiency. Table S3 presents the fiber yield and gum content analyses of the untreated and treated samples (Figure 7a,b). The highest solid residue was found in sample CU120-1, whereas increasing temperature and reaction time led to reduced solid residues. At 160 °C and approximately 3 h of reaction time, minimal solid residue was observed; however, SEM morphology (Figure S5d) indicated potential fiber damage possibly due to increased DES penetration and prolonged reaction times. The penetration of choline chloride and urea within the amorphous regions of cellulose molecules likely initiates hydrolysis and glycosidic bond ruptures as the reaction time increases. This phenomenon suggests deeper DES penetration as time progresses, potentially leading to enhanced degumming effects. However, sample CU160-2 exhibited lower solid residue recovery, consistent with the morphological analyses. Conversely, prolonged treatment at higher temperatures (CU160-3) resulted in fiber rupture, as evidenced by the SEM images (Figure S5d). Figure 7b illustrates the chemical composition of the raw materials and various samples, highlighting significant lignin removal while preserving cellulose integrity. The treatment effectively retained cellulose while demonstrating excellent lignin removal capabilities, owing to the alkaline nature of choline chloride- and urea-based DES [69]. The previous studies show that alkalinity amide DES removed lignin by deprotonating phenolic hydroxyl groups and breaking the bond between the lignin units when the CU treatment was conducted [70]. Thus, this proposed study revealed that choline chloride-based deep eutectic solvents can be used for natural fibers, especially FSB fiber pretreatment, which can be used for further value-added applications.

## 4. Conclusions

Traditional methods of degumming natural fibers have several limitations such as lengthy procedures, high energy consumption, negative environmental impact, and low efficiency. This study presents a pioneering approach focusing on employing DES-synthesized degumming of *Firmiana simplex* bark fibers. This research explores the morphologies, chemical compositions, crystallinities, and physical properties of FSB fibers, as well as the effects and mechanisms of different DESs on the dispersion of FSB. The experimental findings reveal that an alkaline amide DES based on choline chloride and urea effectively disrupts the hydrogen bond interaction within FSB fibers by outcompeting chloride ions. Subsequently, the DES deprotonates phenolic hydroxyl groups and cleaves β-O-4 bonds in lignin units, facilitating efficient lignin removal from the fibers. Among the tested DESs, ChCl–urea (CU) demonstrates the highest effectiveness in degumming FSB. The chemical composition analysis highlighted FSB fibers’ composition, including high cellulose content (71.12 ± 1.5%), significant lignin (16.34 ± 1.03%), and minor wax (0.91 ± 0.03%), influencing their hydrophilic properties. The SEM analysis confirmed that CU-treated fibers exhibited smooth surfaces and retained excellent mechanical properties (breaking tenacity: 5.31 ± 0.08 cN/dtex, linear density: 6.11 ± 0.1 dtex). The optimal degumming conditions involve a precise processing temperature of 160 °C and a controlled reaction time of 2 h, resulting in the most favorable outcomes. The utilization of CU-based DES proves efficient in removing gummy constituents from *Firmiana simplex* bark fibers, enabling the production of refined and dry fibers. This novel approach not only enhances degumming efficiency but also contributes to sustainability by reducing energy consumption and environmental impact. This study presents a straightforward and environmentally friendly degumming method for *Firmiana simplex* bark, offering significant potential to enhance the overall quality and usability of the resulting fibers. Our findings mark a significant advancement in sustainable fiber-processing technologies, highlighting the potential for a broader application of DESs in natural-fiber degumming.

## Data Availability

The data presented in this study are available within this article.

## Supplementary Materials

The following supporting information can be downloaded at: https://www.mdpi.com/article/10.3390/polym16152112/s1, Figure S1. Water retting process to extract short fibers, Figure S2. Unpeeled fibers from raw bark (a), treated with choline chloride and urea (b), choline chloride and oxalic acid (c), choline chloride and lactic acid (d), and choline chloride and ethylene glycol (e), Figure S3. Chopped bark (a), treated with choline chloride and urea (b), choline chloride and ethylene glycol (c), choline chloride and oxalic acid (d), and choline chloride and lactic acid (e), Figure S4. Degumming of Phoenix tree bark, water-retted bark (a), CLA pretreated (b), CE pretreated (c), CO pretreated (d), and CU pretreated (e), Figure S5. SEM morphology of raw material (a), fibers after CU-based DES treatment with CU120-1 (b), CU160-2 (c), and CU160-3 (d), Table S1. Comparison of the chemical composition of various sources of fibers, Table S2. Mechanical properties of different pretreated samples, Table S3. Fiber yield and residual gum content of CU-based pretreated fiber samples (see [71,72,73,74,75,76,77]).
