# Supplementary material for: Maximizing Degumming Efficiency for Firmiana simplex Bark Using Deep Eutectic Solvents
Source: Polymers (Basel). 2024 Jul 24;16(15):2112. doi: 10.3390/polym16152112 (PMC11314601; doi:10.3390/polym16152112)
Supplement: Supplementary file 1 [file polymers-16-02112-s001.zip › polymers-3080313-supplementary.pdf]

Maximizing Degumming Efficiency for *Firmiana simplex* Bark Using Deep Eutectic Solvents

Amjad Farooq [1,2], Muhammad Tauseef Khawar [3], Zongqian Wang [2,*], Mingwei Tian [1,*] and Muhammad Mushtaq [4]

[1] School of Textile and Garment, Qingdao University, Qingdao City 266071, China; amjad@ahpu.edu.cn
[2] School of Textile and Garment, Anhui Polytechnic University, Wuhu 241000, China
[3] School of Engineering and Technology, National Textile University Faisalabad, Faisalabad 37610, Pakistan; tauseefkhawar@ntu.edu.pk
[4] School of Art and Design, National Textile University Faisalabad, Faisalabad 37610, Pakistan;
mushtaqmalik@ntu.edu.pk
* Correspondence: wzqian@ahpu.edu.cn (Z.W.); mwtian@qdu.edu.cn (M.T.)

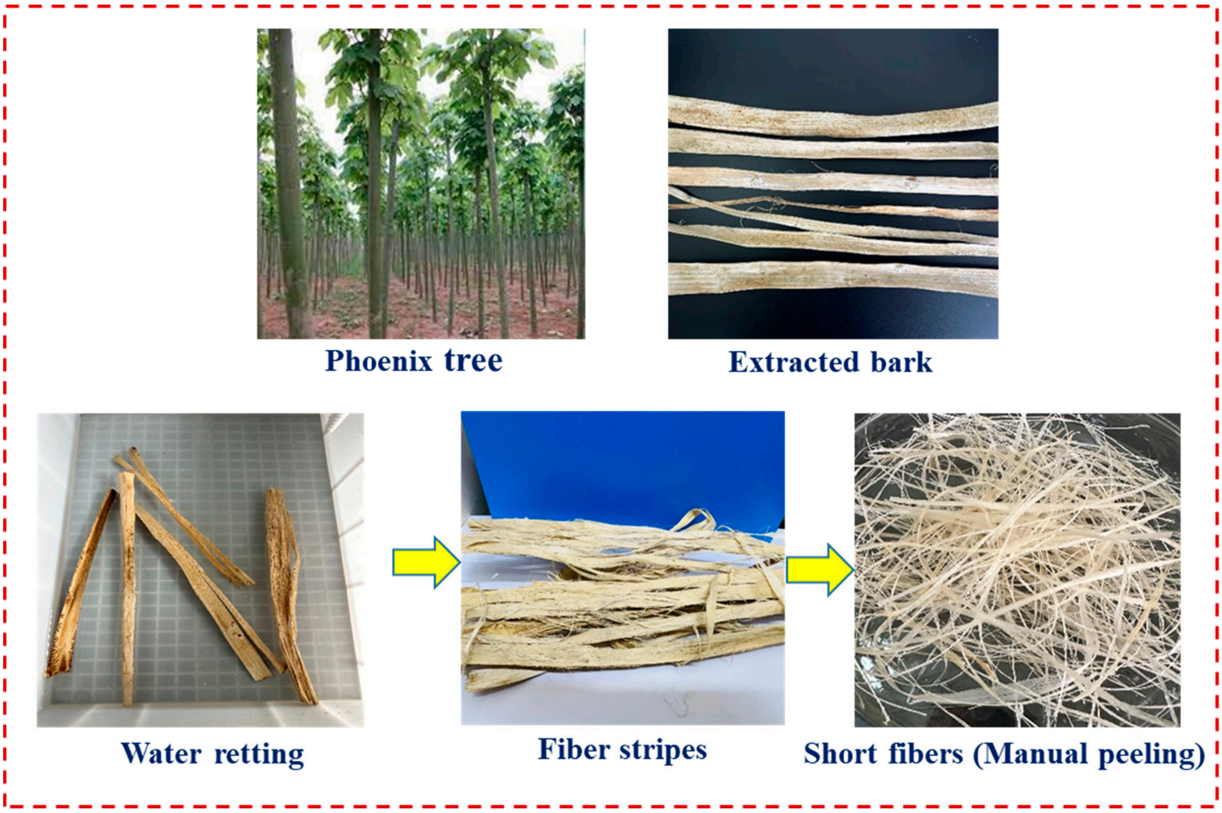

Figure S1. Water retting process to extract short fibers

Chemical Compositional analysis

The raw bark collected from the farmers were sundried for days. Sundried samples after chopped into small pieces about 2 to 3 cm in length.

The extractive percentage was evaluated by taking dried raw bark fiber samples (2.5 g) into a cellulose thimble. Using a Soxhlet extractor, 150 mL of acetone was taken as the extraction solvent. The boiling and rising stages were carefully maintained at 70 °C and 25 minutes, respectively, for a total extraction duration of 4 hours. After extraction, the sample was air-dried at room temperature for a few minutes. It was then placed in a convection oven at 105°C until a constant weight was achieved. The percentage of extractives content (w/w) was determined by calculating the difference in weight between the raw extractive biomass and the extractive-free biomass.

Hemicellulose content percentage was evaluated by taking extracted dried biomass (1 g) into an Erlenmeyer flask (250 mL). Next, 150 mL of 500 mol/m^3 NaOH was added to the flask. The mixture was then boiled for 3.5 hours with distilled water. After cooling, the mixture was filtered using vacuum filtration and washed until it reached a neutral pH. The residue was then dried to a constant weight at 105°C in a convection oven. The hemicellulose content (% w/w) of the dry biomass was determined by calculating the difference in weight before and after the treatment, as referenced in sources.

To analyze lignin content, dried extracted raw biomass (0.3 g) was placed in glass test tubes and treated with 72% H_2SO_4 (3 mL). The sample was kept at room temperature for 2 hours, with stirring of the solution done after every 30 minutes to ensure complete hydrolysis. Afterward, distilled water (84 mL) was added, and the mixture underwent a second hydrolysis step in an autoclave for 1 hour at 121°C. After cooling to room temperature, the hydrolysate was filtered using vacuum filtration through a filtering crucible. The acid-insoluble lignin was determined by drying the residue at 105°C and accounting for ash content by burning the hydrolyzed samples at 575°C in a muffle furnace. The acid-soluble lignin fraction was measured by determining the absorbance of the acid-hydrolyzed samples at 320 nm. The total lignin content was calculated as the sum of the acid-insoluble lignin and the acid-soluble lignin, as referenced in the source. Finally, the cellulose content (% w/w) was determined by subtracting the combined weights of extractives, hemicellulose, lignin, and ash from the total biomass weight. This method assumes that these components, along with cellulose, make up the entire biomass composition, as referenced in sources

Table S1. Comparison of the chemical composition of various sources of fibers

Fiber Source	Cellulose (%)	Hemicellulose (%)	Lignin (%)	References
Corn hubs	45	35	15	[1]
Ficus natalensis bark	43.5±1	24.5±0.8	19.5±0.9	[2]
Hardwood	43-47	25-35	16-24	[1]
Flax	71	18.6–20.6	2.2	[3]
Ficus racemosa	72.36	11.21	10.45	[4]
Grewia monticola	55.74	14.65	15.39	[5]
Hibiscus tiliaceus	58.63	18	23.35	[6]
Napier grass	45.66	33.67	20.60	[7]
Pithecellobium dulce	75.15	10.23	12.14	[8]
Prosopis juliflora	61.65	16.14	17.11	[9]
Prosopis juliflora	61.65	16.14	17.11	[10]
Firmiana simplex bark	71.12 ± 1.5	13.11 ± 0.9	16.64 ± 1.03	Present study

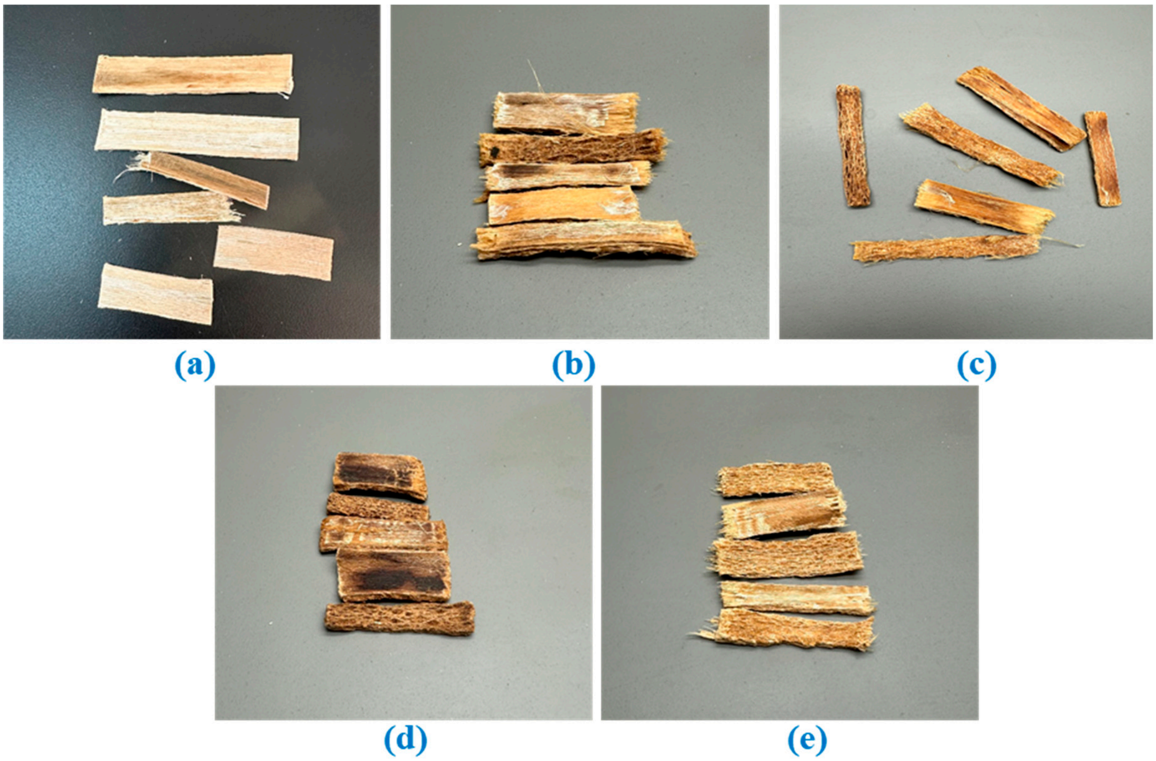

Figure S2. Unpeeled fibers from raw bark (a), treated with choline chloride and urea (b), choline chloride and oxalic acid (c), choline chloride and lactic acid (d), and choline chloride and ethylene glycol (e)

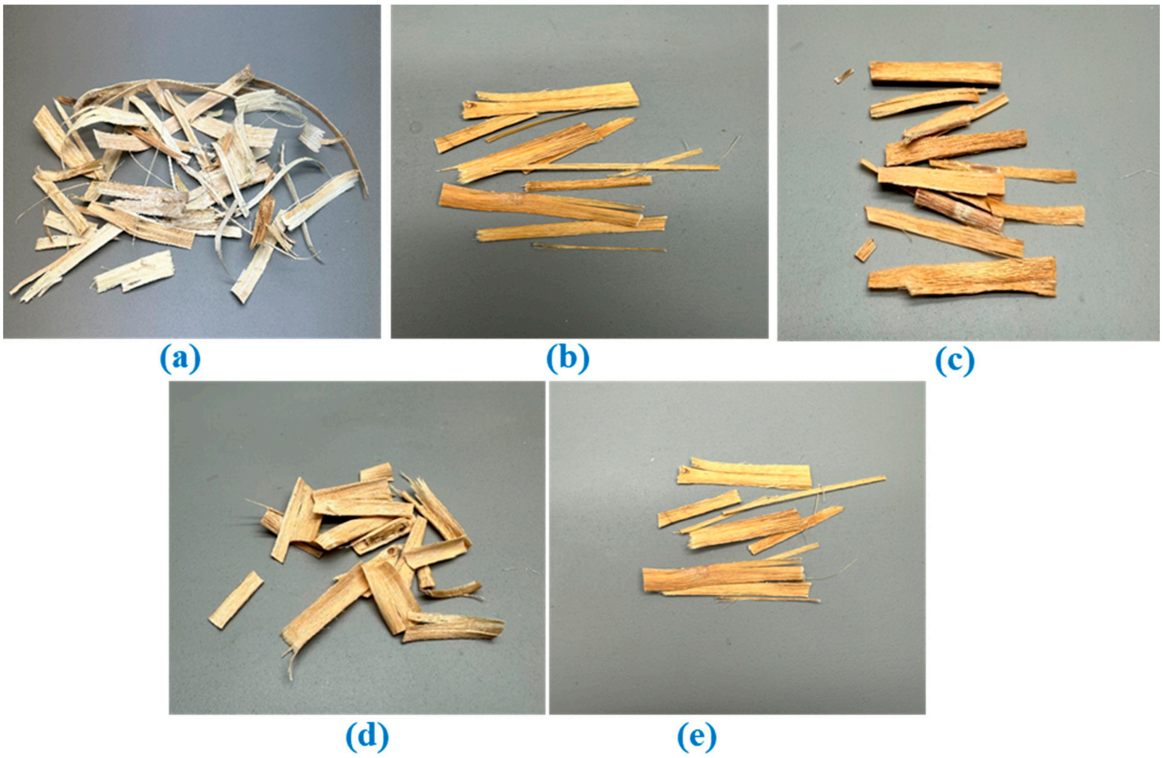

Figure S3. Chopped bark (a), treated with choline chloride and urea (b), choline chloride and ethylene glycol (c), choline chloride and oxalic acid (d), and choline chloride and lactic acid

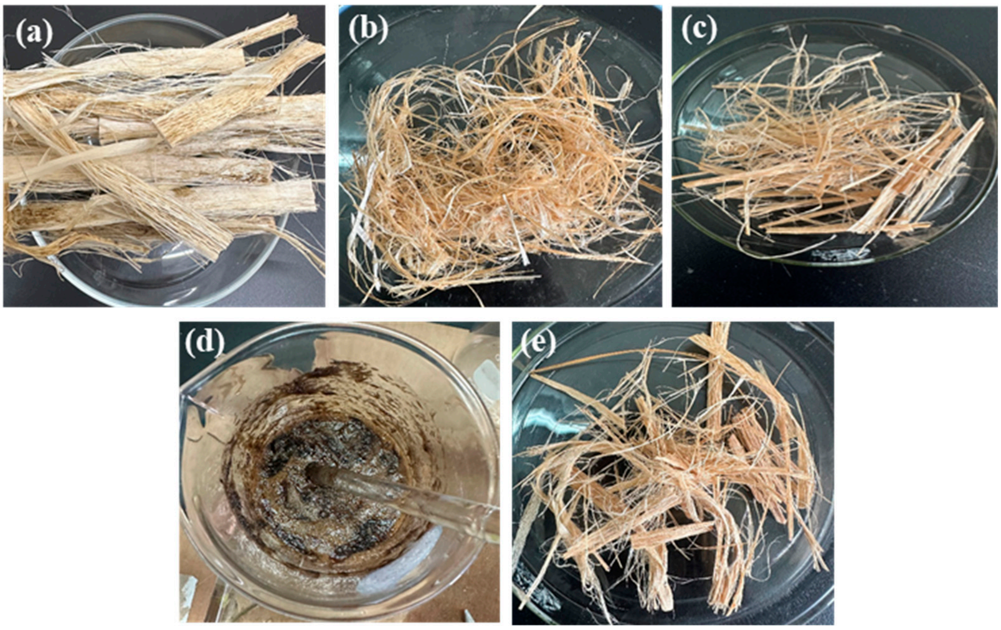

Figure S4. Degumming of Phoenix tree bark, water-retted bark (a), CLA pretreated (b), CE pretreated (c), CO pretreated (d), and CU pretreated (e).

Table S2. Mechanical properties of different pretreated samples

Samples	Linear density (dtex)	Breaking strength (cN/dtex)	Elongation at the break (%)
CU	6.11 ± 0.1	5.31± 0.08	1.98 ± 0.05
EG	6.98 ± 0.1	4.91± 0.07	1.13 ± 0.05
CLA	7.03 ± 0.1	3.40 ± 0.07	1.09 ± 0.05

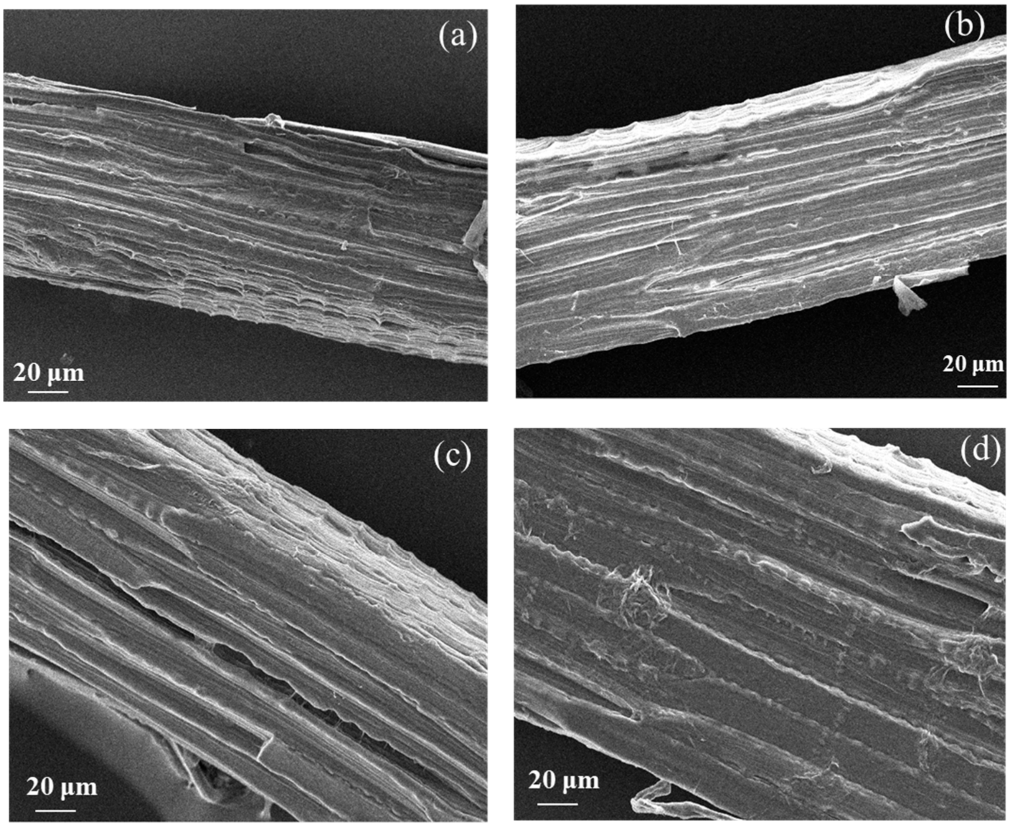

Figure S5. SEM morphology of raw material (a), fibers after CU-based DES treatment with CU120-1 (b), CU160-2 (c), and CU160-3 (d).

Table S3. Fiber yield and residual gum content of CU-based pretreated fiber samples

Samples	Fiber yield（%）	Residual gum content (%)	Remarks
FSB Fiber	/	26.67	Normal
CU 120-1	67.3%	9.40%	Normal
CU 120-2	70.5%	5.54%	Normal
CU 120-3	70.41%	3.74%	Normal
CU 140-1	69.7%	8.34%	Normal
CU 140-2	72.6%	3.21%	Normal
CU 140-3	68.4%	2.98%	Normal
CU 160-1	70.3%	6.09%	Normal
CU 160-2	72.7%	3.02%	Normal
CU 160-3	68.1%	2.89%	DES started to boil

References

1. J. Credou, T. Berthelot, Cellulose: from biocompatible to bioactive material, Journal of Materials Chemistry B 2(30) (2014) 4767-4788.
2. A. Farooq, M. Li, A. Alasood, A. Farooq, M. Ashraf, M.K. Patoary, L. Liu, Novel pretreatment performance evaluation for cellulose nanofibrils extraction from Ficus natalensis barkcloth, Journal of Polymers and the Environment (2022) 1-13.
3. O. Faruk, A.K. Bledzki, H.-P. Fink, M. Sain, Biocomposites reinforced with natural fibers: 2000–2010, Progress in polymer science 37(11) (2012) 1552-1596.
4. P. Manimaran, K. Solai Senthil Kumar, M.J.J.o.N.F. Prithiviraj, Investigation of physico chemical, mechanical and thermal properties of the albizia lebbeck bark fibers, 18(8) (2021) 1151-1162.
5. M. Almeshaal, S. Palanisamy, T.M. Murugesan, M. Palaniappan, C.J.J.o.N.F. Santulli, Physico-chemical characterization of Grewia Monticola Sond (GMS) fibers for prospective application in biocomposites, 19(17) (2022) 15276-15290.
6. W.A. Wirawan, M.A. Choiron, E. Siswanto, T.D.J.J.o.N.F. Widodo, Morphology, structure, and mechanical properties of new natural cellulose fiber reinforcement from waru (Hibiscus tiliaceus) bark, 19(15) (2022) 12385-12397.
7. K.O. Reddy, C.U. Maheswari, M. Shukla, A.V.J.M.l. Rajulu, Chemical composition and structural characterization of Napier grass fibers, 67(1) (2012) 35-38.
8. P. Manimaran, M. Sanjay, P. Senthamaraikannan, B. Yogesha, C. Barile, S.J.J.o.N.F. Siengchin, A new study on characterization of Pithecellobium dulce fiber as composite reinforcement for light-weight applications, (2018).
9. S. Saravanakumar, A. Kumaravel, T. Nagarajan, P. Sudhakar, R.J.C.p. Baskaran, Characterization of a novel natural cellulosic fiber from Prosopis juliflora bark, 92(2) (2013) 1928-1933.
10. S. Saravanakumar, A. Kumaravel, T. Nagarajan, P. Sudhakar, R. Baskaran, Characterization of a novel natural cellulosic fiber from Prosopis juliflora bark, Carbohydrate polymers 92(2) (2013) 1928-1933.
